# The scavenging capacity of DMBT1 is impaired by germline deletions

**DOI:** 10.1007/s00251-017-0982-x

**Published:** 2017-03-31

**Authors:** Floris J. Bikker, Caroline End, Antoon J. M. Ligtenberg, Stephanie Blaich, Stefan Lyer, Marcus Renner, Rainer Wittig, Kamran Nazmi, Arie van Nieuw Amerongen, Annemarie Poustka, Enno C.I. Veerman, Jan Mollenhauer

**Affiliations:** 1 Department of Oral Biochemistry, Academic Centre for Dentistry Amsterdam, University of Amsterdam and VU University Amsterdam, Gustav Mahlerlaan 3004, 1081LA Amsterdam, Netherlands; 2 Division of Molecular Genome Analysis, German Cancer Research Center, Heidelberg, Germany; 3 Molecular Oncology, University of Southern Denmark, Odense, Denmark

**Keywords:** SRCR domain, Genetic polymorphism, Pathogen binding, Microbial defense

## Abstract

The Scavenger Receptor Cysteine-Rich (SRCR) proteins are an archaic group of proteins characterized by the presence of multiple SRCR domains. They are membrane-bound or secreted proteins, which are generally related to host defense systems in animals. Deleted in Malignant Brain Tumors 1 (DMBT1) is a SRCR protein which is secreted in mucosal fluids and involved in host defense by pathogen binding by its SRCR domains. Genetic polymorphism within DMBT1 leads to DMBT1-alleles giving rise to polypeptides with interindividually different numbers of SRCR domains, ranging from 8 SRCR domains (encoded by 6 kb *DMBT1* variant) to 13 SRCR domains (encoded by the 8 kb *DMBT1* variant). In the present study, we have investigated whether reduction from 13 to 8 amino-terminal SRCR domains leads to reduction of bacterial binding. The 6 kb variant bound ~20–45% less bacteria compared to the 8 kb variant. These results support the hypothesis that genetic variation in DMBT1 may influence microbial defense.

## Introduction

The Scavenger Receptor Cysteine-Rich (SRCR) proteins are an archaic group of highly conserved proteins in animals (Aruffo et al. 1997; Freeman et al. 1990; Muller et al. 1999; Pahler et al. 1998; Resnick et al. 1994). The SRCR-superfamily is comprised of cell membrane-anchored proteins as well as secretory proteins. SRCR proteins are characterized by the presence of multiple SRCR domains. SRCR domains are approximately 110 amino acids long and are classified into groups A and B based on the number of conserved cysteine residues (six for group A, eight for group B) (Aruffo et al. 1997; Resnick et al. 1994). Generally, SRCR proteins, e.g., the macrophage scavenger receptor, Mac2-binding protein, CD5, CD6, and WC1, have generally been implicated into host defense systems (Aruffo et al. 1997; Aruffo et al. 1991; Elomaa et al. 1998; Freeman et al. 1990; Gough and Gordon 2000; Holmskov et al. 1999; Ligtenberg et al. 2001; Prakobphol et al. 2000; Tino and Wright 1999).

*Deleted in Malignant Brain Tumors 1* (*DMBT1*) at chromosome 10q25.3-q26.1 is a member of the group B SRCR superfamily (Holmskov et al. 1997; Holmskov et al. 1999; Ligtenberg et al. 2001; Mollenhauer et al. 1997; Prakobphol et al. 2000). DMBT1 is composed of 13 highly homologous SRCR domains (Aruffo et al. 1997; Hohenester et al. 1999), separated by SRCR-interspersed domains (SIDs), two CUB (C1r/C1s Uegf Bmp1) domains (Bork and Beckmann 1993; Romero et al. 1997), separated by a 14th SRCR domain, and a Zona Pellucida domain (Jovine et al. 2002; Sinowatz et al. 2001). DMBT1 is expressed in saliva (DMBT1^SAG^) and other mucosal fluids along the gastro-intestinal tract, in the lungs (DMBT1^GP340^), and amniotic fluid (Holmskov et al. 1997; Holmskov et al. 1999; Ligtenberg et al. 2004; Mollenhauer et al. 1997; Prakobphol et al. 2000; Reichhardt et al. 2014). In saliva, the concentration of DMBT1^SAG^ is approximately 20 μg/ml (Sonesson et al. 2011). DMBT1 plays various roles in innate immunity, e.g., by activating the MBL-mediated lectin pathway of the complement system (Gunput 2016); it binds to surfactant proteins A and D (Holmskov et al. 1997; Tino and Wright 1999), IgA (Ligtenberg et al. 2004), MUC5B (Thornton et al. 2001), C-type lectin receptors DC-SIGN and Langerin (Boks et al. 2016), influenza virus and HIV (Hartshorn et al. 2003; Stoddard et al. 2007), the dental hard tissue (Bikker et al. 2013), and a wide spectrum of bacteria (Bikker et al. 2004; Bikker et al. 2002; Leito et al. 2008; Madsen et al. 2010). In particular, the SRCR/SID region in DMBT1 appears to play a dominant role in bacterial binding (Bikker et al. 2004; Bikker et al. 2002; Brittan and Nobbs 2015; Kukita et al. 2013).

We have unraveled genetic polymorphism within DMBT1 (Mollenhauer et al. 1999; Mollenhauer et al. 2002b). This results in DMBT1 alleles encoding polypeptides that have different numbers of SRCR domains within the SRCR/SID region, ranging from 8 to 13. This SRCR/SID region does not include the carboxy-terminal SRCR domain, which is located in between the CUB domains. This 14th SRCR domain does not show bacterial-binding activity (Holmskov et al. 1997). Based on analogies to mucins, we postulate that these polymorphisms may lead to a differential efficacy in mucosal protection (Kohlgraf et al. 2003; Mollenhauer et al. 2001; Mollenhauer et al. 2000; Polley et al. 2015).

In the present study, we have selected individuals homozygous for either 8 or 13 SRCR domains and showed a corresponding reduction of the protein size of DMBT1^SAG^. We consistently observed that, compared to wild-type DMBT1 (8 kb, 13 SRCR domains in the SID/SRCR region), the short DMBT1variant (6 kb, 8 SRCR domains in the SID/SRCR region) displayed 30–45% reduced capacity in binding to bacteria in vitro.

## Materials and methods

### Southern blotting

Genomic DNA from healthy volunteers (ethnic background: Caucasian) was extracted from peripheral blood leukocytes (PBL) according to standard procedures. Collection of blood samples and genetic analyses were approved by the ethics committee of the University of Heidelberg. Twenty-microgram of genomic DNA was digested overnight with the restriction enzyme RsaI (Roche Diagnostics; 10 U/μg DNA), ethanol-precipitated, and resuspended in a total volume of 40 μl H_2_O. The digested DNA was separated for 20–22 h on 1.2% (*w*/*v*) agarose gels at 45 V. From this point on, everything was exactly done as described previously (Mollenhauer et al. 1999), with the exception that exclusively probe DMBT1/sr1sid2 was used.

### Bacteria

S*treptococcus mutans* (Ingbritt), *Streptococcus gordonii* (HG222) and *Escherichia coli* (F7) were cultured on blood agar plates under anaerobic conditions with 5% CO_2_ at 37 °C for 24 h. Subsequently, single colonies were cultured in Todd Hewitt medium and in Luria Broth (Oxoid, Hampshire, United Kingdom) for *S. mutans, S. gordonii*, and *E. coli*, respectively, overnight in air/CO_2_ (19:1), at 37 °C. Cells were harvested and washed twice in TTC buffer (TBS-Tween-Calcium buffer: TBS, 150 mM NaCl, 10 mM Tris-HCl, pH 7.4; 0.01% (*v*/v) Tween 20 (polysorbate, Merck-Schuchardt, Germany); 1 mM calcium). *Helicobacter pylori* (NCTC 11637) was cultured on selective Dent plates (Oxoid) at 37 °C for 72 h. *H. pylori* was harvested by wiping off the plates and washed twice in NTC buffer (100 mM Sodium acetate, pH 4.2, 0.01% (*v*/v) Tween 20, supplemented with 1 mM calcium). Bacteria were diluted in buffer to a final OD_700_ of 0.5, corresponding with approximately 5 × 10^8^ cells/ml.

### Collection of DMBT1^SAG^ and determination of relative concentration

Human parotid saliva was collected from healthy donors with a Lashley cup, under stimulation by chewing on sugar-free chewing gum. Collection and use of saliva was approved by the ethics committee of the University of Heidelberg. Twenty-five milliliters of parotid saliva was kept on ice water for 30 min, to promote the formation of a precipitate. This precipitate was collected by centrifugation at 5000x*g* at 4 °C for 20 min. The resulting pellet was dissolved in 2.5 ml TBS. The pellet was approximately tenfold enriched in DMBT1^SAG^ (~200 μg/ml), designated as crude DMBT1^SAG^.

For qualitative adhesion assays with DMBT1^SAG^, crude DMBT1^SAG^ samples from saliva donors (A and B) were titrated against monoclonal antibody (mAb) DMBT1h12. The antibody recognizes a non-repetitive, non SRCR domain, peptide epitope (amino acid 26–40), which is present within all known DMBT1 variants and locates outside the region that shows germline deletions (Fig. 2a) (Stoddard et al. 2007). High affinity microtiter plates (Greiner-F, Polysorp, Nunc, Kamstrup, Denmark) were coated with crude DMBT1^SAG^, in coating buffer (100 mM sodium carbonate, pH 9.6) for 2 h at 37°C. This incubation and all the following steps were carried out in a volume of 100 μl per well at room temperature, and all washes and incubations were carried out in TTC Buffer. Plates were incubated for 1 h with 1:500 mAb DMBT1h12. After washing, the plates were incubated at 37 °C for 1 h with a rabbit anti-mouse IgG-HRP conjugate (dilution 1:2000 in TTC; DAKO A/S, Denmark). Subsequent to three washes with TTC, 100 μl TMB-solution (3,3′,5,5′-Tetramethyl-benzidine; 125 μg/ml in citrate buffer pH 4.5 with 0.05% *v*/v H_2_O_2_) was added, and after incubation at RT for 10–15 min, the reaction was stopped by the addition of 50 μl 2 M sulphuric acid per well. The absorbance was read at 405 nm on a Dynatech MR7000 plate reader (Billington, UK). The results of the ELISA were used to dilute the different DMBT1^SAG^ samples to obtain solutions containing comparable concentrations of the unique DMBT1 epitope, which is not the SRCR-epitope. For the adhesion assay, these equalized DMBT1^SAG^ solutions were coated onto microtiterplates.

### Adhesion assays

Bacterial adhesion was examined using a microtiter plate method based on labeling of microorganisms with cell-permeable DNA-binding probes (Bikker et al. 2004; Bikker et al. 2002). Microtiterplates Fluotrac 600 (Greiner, Recklinghausen, Germany) were coated with equal amounts of crude SAG from donors A and B. For this, the samples were dissolved in coating buffer (100 mM sodium carbonate, pH 9.6) and diluted serially. Bovine Serum Albumin (BSA, Sigma-Aldrich, Zwijndrecht, the Netherlands), coated from 1 to 0.01%, was used a control. This experiment was conducted after the adhesion experiments using DMBT1^SAG^. After incubation at 4 °C for 16 h, plates were washed twice with TTC. In case of *H. pylori*, plates were washed with NTC. Subsequently, 100 μl of a bacterial suspension (5 × 10^8^ bacteria/ml) were added to each well and incubated for 2 h at 37 °C. Plates were washed three times with TTC, or NTC for *H. pylori*, using a plate washer (Mikrotek EL 403, Winooski, VT). Bound bacteria were detected using 100 μl/well of 1 mM SYTO-9 solution (Molecular Probes, Leiden, The Netherlands), a cell-permeable fluorescent DNA-binding probe. Plates were incubated in the dark for 15 min at ambient temperature and washed three times with 0.1% Tween 20. Fluorescence was measured in a fluorescence microtiter plate reader (Fluostar Galaxy, BMG Laboratories, Offenburg, Germany) at 488 nm excitation and 509 nm emission wavelength. The experiments were performed four times, in duplicate.

### SDS-PAGE and western blotting

Samples were incubated at 100 °C for 10 min in sample buffer containing 15 mM Tris-HCl, pH 6.8, 0.5% SDS, 2.5% glycerol, 25 mM dithiothreitol and 0.05% bromophenol blue. SDS-PAGE was conducted on a Pharmacia Phast System (Pharmacia-LKB, Uppsala, Sweden) using 7.5% polyacrylamide gels, according to the manufacturers protocol. The approximate concentration DMBT1^SAG^ loaded on SDS-PAGE was 2 μg.

Western blotting was performed as described before (Ligtenberg et al. 2001). Nitrocellulose membranes were incubated with mAb DMBT1h12 antibodies. Bound antibodies were detected with alkaline phosphatase-conjugated to rabbit anti-mouse immunoglobulins (DAKO, Glostrup, Denmark) using 5-bromo-4-chloro-3-indolyl-phosphate (X-P) and nitro blue tetrazolium chloride (NBT) (Boehringer Mannheim, Germany) as substrate.

### Statistical analysis

The mean bacteria binding activity of the 6 and 8 kb variant was compared with Mann-Whitney U tests, using IBM SPSS Statistics for Windows version 20.0 (IBM Corp, Armonk NY. USA). *P* values <0.05 were considered statistical significant.

## Results

### Determination of interindividual polymorphism of DMBT1

**Fig. 1:**
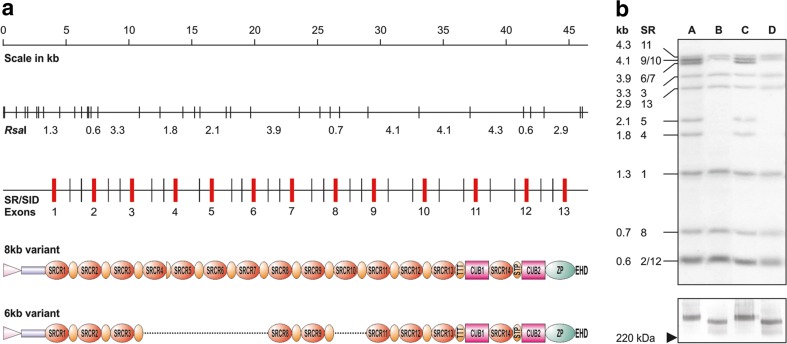
DMBT1 polymorphism leads to different length DMBT1 polypeptides. **a** Schematical presentation of the exon-intron structure within the relevant region of *DMBT1* with resulting *Rsa*I restriction fragment sizes depicted below*. Gray boxes* denote restriction fragments hybridizing with the probe *DMBT1*/sr1sid2. SR exons coding for scavenger receptor cysteine-rich domains. The hypothetical configurations within the proteins are depicted below. In the carboxy-terminal part of the protein resulting from the deleted allele, it cannot be discerned between a loss of either SR9, SR10, or SR11. Only one of the possibilities is shown. *Pink triangle* signal peptide, *blue box* motif without homology, *SRCR* scavenger receptor cysteine-rich domain, *CUB* C1r/C1s-Uegf-Bmp1 domains, *ZP zona pellucida* domain. *EHD* Ebnerin-homologous domain, *orange ovals* SRCR interspersed domains (SIDs), *TTT* and *STP* are threonine and serine-threonine-proline-rich domains, respectively. **b** *Top panel* Southern blot analysis of the *DMBT1* genomic configuration in four individuals (A–D) selected from the panel. Band sizes and exons locating on the restriction fragments are depicted at the *left*. *Bottom panel* Western blot analysis of DMBT1^SAG^ protein sizes in the partially purified and concentration-adjusted saliva samples of the four probands. The *arrowhead* denotes the position of the 220-kDa marker band. DMBT1^SAG^ was collected from saliva donors that were homozygous for *DMBT1*/8 kb (donors A and C), homozygous for *DMBT1*/6 kb (donors B and D). Crude DMBT1^SAG^ from the four donors samples were separated on 7.5% polyacrylamide gels, transferred to nitrocellulose and immunoblotted with mAb DMBT1H12 . Lane 1 donor A, lane 2, donor B; lane 3, donor C; lane 4, donor D. DMBT1^SAG^ of donors A and C migrated at a position corresponding to an apparent molecular mass of approximately 340 kDa. DMBT1^SAG^ of donors B and D runs at a position corresponding to approximately 255 kDa

Some years ago, we discovered genetic polymorphism within DMBT1 (Mollenhauer et al. 2002a; Mollenhauer et al. 1999; Mollenhauer et al. 2002b) (Fig. 1). This lead us to hypothesize that this polymorphism results in a differential efficacy in mucosal protection. In order to answer this hypothesis, we first screened 200 persons for genetic DMBT1 polymorphism. We found two persons (donors A and C) that were homozygous for a small *DMBT1* variant with 8 SRCR domains in the SID/SRCR region (encoded by the 6 kb *DMBT1* variant). Furthermore, we found two persons (donors B and D) that were homozygous for a large *DMBT1* variant with 13 SRCR domains in the SID/SRCR region (encoded by the 8 kb *DMBT1*variant) (Fig. 1).

**Fig. 2:**
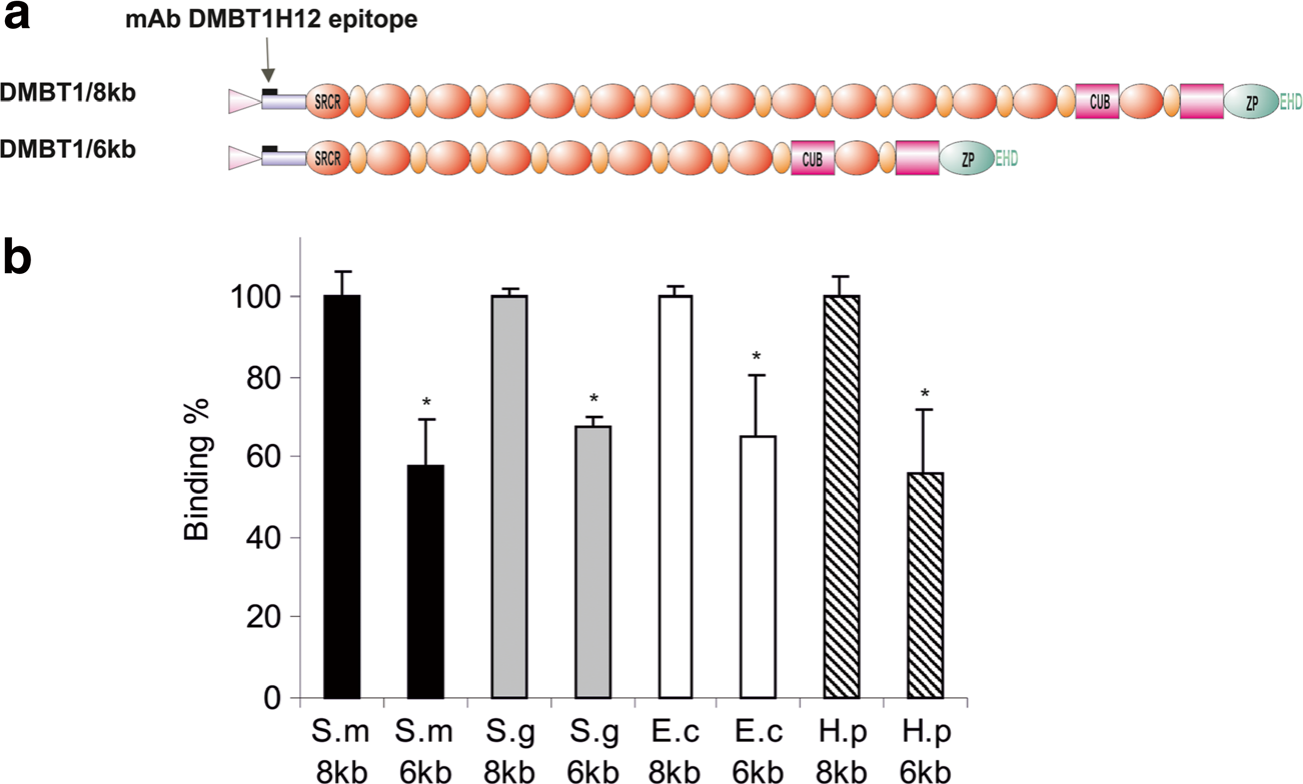
Bacterial binding is dependent on DMBT1 polymorphism. **a** Domain structure of the DMBT1-variant expressed from the large *DMBT1* allele (*DMBT1*/8 kb, 13 SRCR domains within the SRCR/SID region) and the small *DMBT1* allele (*DMBT1*/6 kb, 8 SRCR domains within the SRCR/SID region). *Pink* triangle leader peptide, *blue box* sequence contains unique epitope for mAb DMBT1H12, *red ovals* SRCR domains, *orange ovals*, *SRCR* interspersed domains (SIDs), *purple boxes* C1r/C1s-Uegf-Bmp1 domains, *green oval* zona pellucida domain, *EHD* Ebnerin-Homologous Domain. **b** Bacterial binding to DMBT1/8 kb and DMBT1/6 kb (A) was *semi-*quantified using *S. mutans* (S.m)*, S. gordonii* (S.g.)*, E. coli* (E.c.), and *H. pylori* (H.p.). Relative to the wild type DMBT1^SAG^/8 kb we found, on a molecular base, a decrease in bacterial binding to DMBT1^SAG^/6 kb for all bacteria tested. *Error bars* represent the standard error of the mean (SEM), *P* ≤ 0.05

Now that DMBT1 polymorphism was found on a genetic level, we wanted to confirm these findings on the polypeptide level. For this, we collected DMBT1^SAG^ from the four donors and analyzed protein size by SDS-PAGE and subsequent Western analysis, using mAb DMBT1h12 for immunodetection. In SDS-PAGE DMBT1^SAG^ of donors A and C migrated to a position corresponding with an apparent molecular mass of approximately 255 kDa. DMBT1^SAG^ of donors B and D migrated in SDS-PAGE to a position corresponding with an apparent molecular mass of approximately 340 kDa. These data agreed with the genetic analysis (Fig. 1), revealing that on the one hand donors A and C express the “short” DMBT1^SAG^ of 255 kDa, which putatively contains 8 SRCR domains in the SID/SRCR region. On the other hand, donors B and D express the “long” DMBT1^SAG^ isoform, which putatively contains the SID/SRCR region with 13 SRCR domains (Figs. 1 and 2a).

### DMBT1 polymorphism leads to differential bacterial binding

To examine whether this polymorphism affects biological relevant function of DMBT1^SAG^, we compared the bacteria-binding properties of two different genetic variants from donors A (8 kb) and B (6 kb). First, the DMBT1^SAG^ content of the various preparations was quantified in ELISA using mAbDMBT1h12. Then, each sample was diluted so that the preparations matched each other in epitope concentration, as was verified by western analysis using mAb DMBT1h12 (Fig. 1b). The solutions subsequently were used to coat microtiter plate wells. Equal coating densities of the different DMBT1 preparations were confirmed by ELISA.

The wells coated with DMBT1^SAG^ were incubated with various bacterial species, including *S. mutans*, *S. gordonii*, *E. coli*, *and H. pylori* for 1 h. After washing and addition of the fluorogenic probe, the number of adhering cells was quantified by fluorescence (Fig. 2). The results indicated that wells coated with the short variant DMBT1^SAG^ 6 kb bound, on a molar base, significantly less bacteria, than those coated with the long-variant DMBT1^SAG^ 8 kb. The relative difference in binding for the bacteria tested were 42.4% (+/−11.7) for *S. mutans* (*P* = 0.016), 32.5% (+/−2.7) for *S. gordonii* (*P* = 0.031), 44.3% (+/−15.5) for *E. coli* (*P* = 0.029) and 35.1% (+/−16.0) for *H. pylori* (*P* = 0.032) (Fig. 2b). No binding was observed on the BSA coated microplates (data not shown).

## Discussion

In this study, we demonstrated that genetic polymorphism, i.e., a reduction of the tandem repeat of the SRCR domains and SIDs of DMBT1, results in a corresponding reduction of the protein size (Fig. 1 and 2a). In a previous study, using an ELISA based adherence assay, was shown that DMBT1^SAG^ displays binding to a wide variety of bacteria. Using this assay, we quantified bacterial binding of DMBT1 variants isolated from donors with different genotypes. Based on the assumption that a single SRCR domain contains a single bacterial binding site, located in a putative cleft (Bikker et al. 2004; Bikker et al. 2002; Muller et al. 1999), 8 SRCR within the SRCR/SRCR region domains should theoretically contain 38% less binding capacity than 13 SRCR domains.

We observed that, compared to wild-type DMBT1 (8 kb, 13 SRCR domains in the SID/SRCR region), the short DMBT1variant (6 kb, 8 SRCR domains in the SID/SRCR region) displayed an about 30–45% reduced capacity to bind Gram-positive and Gram–negative bacteria in vitro. This suggests that genetic polymorphism of DMBT1 impairs its protective functions, as supported by complete inactivation in *DMBT1*-knockout mice (Renner et al. 2007). Genetic polymorphism within *DMBT1* has been described extensively and appears to be exceptionally high (Mollenhauer et al. 2000; Mollenhauer et al. 1999; Polley et al. 2015). It has also been reported for other SRCR proteins as well, and seems to be an overall characteristic feature of members of the SRCR superfamily. Genetic polymorphism for SRCR proteins has been reported, e.g., for human CD5 (Padilla et al. 2000), human CD163 sponge Aggregation Receptor (Muller et al. 1999; Pancer 2000), and sheep T19 (Walker et al. 1994). Genetic polymorphism of *DMBT1* was suggested to be linked to functionality, i.e., bacterial binding and hydroxyapatite binding of the polypeptide of DMBT1^SAG^ (Bikker et al. 2013; Bikker et al. 2002). Copy number variants that bound strongly to *S. mutans* and less to hydroxyapatite seemed to be increased in agricultural populations compared to hunter-gatherer populations and ancient hominins (Polley et al. 2015). As *S. mutans* is the causative agent in dental caries; it was speculated that some kind of selection for caries resistance had occurred in agricultural populations. We show that deletion of the SRCR domains not only affects binding to *S. mutans*, but also to other bacteria. Selective pressure from other bacterial infections, which occur more frequently in agricultural populations, may have favored the selection of strong binding copy number variants.

It has to be noted that in this study crude preparations of DMBT1^SAG^ were used for bacterial adhesion analysis.

Although, it cannot be excluded that a variety of proteins such as amylase, proline rich proteins, and s-IgA may have co-precipitated with DMBT1^SAG^; it appears that crude DMBT1^SAG^ shows representative bacteria binding characteristics. In line with earlier studies with purified DMBT1^SAG^ and recombinant DMBT1, crude DMBT1^SAG^ preparations show comparable bacteria binding characteristics (Bikker et al. 2004; Bikker et al. 2002; End et al. 2009; End et al. 2005; Leito et al. 2008; Ligtenberg et al. 2004; Ligtenberg et al. 2001). And, as DMBT1^SAG^ is obtained directly from the parotid gland, the presence of bacteria-binding salivary mucins (MUC7) can be excluded (Veerman et al. 1996).

The present data suggest that the SRCR/SID region defines a complex multi-allele system that represents a possible basis for the variability in human susceptibility to infection as suggested in earlier papers (Mollenhauer et al. 2002a; von Deimling et al. 2000).
